# Umbilical Cord Mesenchymal-Stem-Cell-Derived Exosomes Exhibit Anti-Oxidant and Antiviral Effects as Cell-Free Therapies

**DOI:** 10.3390/v15102094

**Published:** 2023-10-15

**Authors:** Yi Meng, Chengcheng Li, Yicong Liang, Yu Jiang, Haonan Zhang, Jianhua Ouyang, Wen Zhang, Rumei Deng, Qiuping Tan, Xiaolan Yu, Zhen Luo

**Affiliations:** 1 Institute of Medical Microbiology, Jinan University, Guangzhou 510632, China; mengyi@stu2020.jnu.edu.cn (Y.M.); lcc1009@stu2021.jnu.edu.cn (C.L.); liangyicong@stu2017.jnu.edu.cn (Y.L.); 2 State Key Laboratory of Biocatalysis and Enzyme Engineering, Hubei University, Wuhan 430062, China; jy305910669@163.com (Y.J.); zhanghaonan0618@163.com (H.Z.); 3 Foshan Institute of Medical Microbiology, Foshan 528315, China; ouyangjianhua818@163.com (J.O.); bye_cherish_yumy@163.com (R.D.); 4 Guangdong Longfan Biological Science and Technology Company, Foshan 528315, China; zw@gd-longfan.com (W.Z.); qp1019@sina.com (Q.T.); 5 Laboratory of Viral Pathogenesis & Infection Prevention and Control (Jinan University), Ministry of Education, Guangzhou 510632, China

**Keywords:** umbilical cord mesenchymal stem cells (ucMSCs), exosomes, oxidative damage, enterovirus, antiviral activity, cell-free therapy

## Abstract

The oxidative stress induced by the accumulation of reactive oxygen species (ROS) can lead to cell aging and death. Equally, the skeletal muscle usually hosts enteroviral persistent infection in inflammatory muscle diseases. As excellent bioactive products, the exosomes derived from umbilical cord mesenchymal stem cells (ucMSCs) have been proven to be safe and have low immunogenicity with a potential cell-free therapeutic function. Here, exosomes derived from ucMSCs (ucMSC-EXO) were extracted and characterized. In a model of oxidative damage to skin fibroblasts (HSFs) under exposure to H_2_O_2_, ucMSC-EXO had an observable repairing effect for the HSFs suffering from oxidative damage. Furthermore, ucMSC-EXO inhibited mitogen-activated protein kinases (MAPK), c-Jun N-terminal kinase (JNK), and nuclear factor kappa-B (NF-κB) signaling pathways, thereby promoting p21 protein expression while decreasing lamin B1 protein expression, and finally alleviated oxidative stress-induced cell damage and aging. In a model of rhabdomyosarcoma (RD) cells being infected by enterovirus 71 (EV71) and coxsackievirus B3 (CVB3), the ucMSC-EXO enhanced the expression of interferon-stimulated gene 15 (ISG15) and ISG56 to inhibit enteroviral replication, whereafter reducing the virus-induced proinflammatory factor production. This study provides a promising therapeutic strategy for ucMSC-EXO in anti-oxidative stress and antiviral effects, which provides insight into extending the function of ucMSC-EXO in cell-free therapy.

## 1. Introduction

The skin is the first line of defense against external stimuli and consists of the epidermis, dermis, and subcutaneous tissue, of which human skin fibroblasts (HSFs) are the main cellular component of the dermal tissue involved in the recovery of dermal damage [1]. The increase in reactive oxygen species (ROS) induced by an intracellular oxidant such as H_2_O_2_ causes persistent damage to the telomeric and mitochondrial DNA, and leads to a DNA-damage response (DDR). Consequently, the senescence-associated secretory phenotype (SASP) is increased, and the p38 mitogen-activated protein kinases (p38 MAPK), c-Jun N-terminal kinase (JNK), and nuclear factor kappa-B (NF-κB) signaling pathways are activated with the upregulation of IL-2, IL-4, IL-6, IL-8, and TNF-α expression [2,3]. Thus, the skin is inevitably affected by the internal aging process, while various external environmental factors accelerate the development of skin-aging-related diseases.

The local skin and skeletal muscle are linked in the regulation of temperature and circulation [4]. Enteroviruses have been detected in muscle biopsies from patients with chronic inflammatory muscle diseases during infection [5]. Among human enteroviruses, enterovirus 71 (EV71) and coxsackievirus B3 (CVB3) are important causes of hand-foot-and-mouth disease (HFMD) in infants, especially in China, which infect the muscle cells and spread through the muscle, resulting in inflammation [6]. Unfortunately, there are no effective and compatible therapeutic strategies for muscle diseases caused by enteroviral infection. 

Mesenchymal stem cells (MSCs) are pluripotent stromal cells with pluripotent differentiation potential and immunosuppressive properties, obtained from a variety of sources, including the umbilical cord, bone marrow, or adipose tissue, allowing them to promise candidate cells suitable for immune regulation and regeneration [7,8]. Human umbilical cord mesenchymal stem cells (hucMSCs) are an ideal therapeutic approach due to their advantages of easy extraction and expansion, low cost, non-invasive collection procedures, high cellular content, low risk of pathogenic infections, higher proliferation factor, and lower immunogenicity [9,10]. Therefore, hucMSCs and their products are deemed to be widely suitable for use in tissue and cell repair therapies.

Exosomes are endosome-derived extracellular vesicles (EVs) [11] with a diameter of 30–200 nm and originally defined according to their endocytic origin [12,13]. MSC-derived exosomes display multiple biological functions and can be easily used for cell-free therapy in many kinds of diseases [14]. Notably, ucMSC-derived exosomes (ucMSC-EXO) have been proven to be safe and low-immunogenicity, showing considerable immunoregulation and regenerative ability [15]. However, the therapeutic effects and potential mechanisms of ucMSC-EXO on oxidative stress and enteroviral replication are not fully understood. Here, we aim to evaluate the role of ucMSC-EXO in resisting oxidative stress in HSFs and their effect on enterovirus infection in the skeletal muscle.

## 2. Materials and Methods

### 2.1. Cell Culture

The human skin fibroblasts (HSFs), also named human foreskin fibroblasts-1 (HFF-1) (Cat: SCRC-1041), and rhabdomyosarcoma (RD) cells (Cat: CCL-136) were purchased from the American Type Culture Collection (ATCC) (Manassas, VA, USA), and cultured in high-glucose Dulbecco’s modified Eagle’s medium (DMEM) (Gibco; Grand Island, NY, USA) supplemented with 10% fetal bovine serum (FBS) (Gibco). The umbilical cord mesenchymal stem cells (ucMSCs) (Cat: #7530) were obtained from ScienCell Research Laboratories (Carlsbad, CA, USA) and cultured in a serum-free MSC medium (Cat: NC0103) (Yocon Biotech. Co.; Beijing, China). The cells were maintained at 37 °C in a humidified atmosphere containing 5% CO_2_ and passaged every 3–5 days. The other exosome donor cells, human embryonic kidney cell line (HEK293T), cardiomyocytes line (AC16), human intestinal cells (HT29), and human brain microvascular endothelial cells (hBMEC), were purchased from the ATCC, which were maintained in the above culture condition. 

### 2.2. Antibodies and Reagents

FITC mouse anti-human CD19 (Cat: 560994), CD34 (Cat: 560942), CD45 (Cat: 560976), CD73 (Cat: 561254), and CD105 (Cat: 561443) and PE mouse anti-human CD90 (Cat: 555596) were purchased from BD Pharmingen (BD Biosciences Systems; San Jose, CA, USA). The rabbit antibodies against phosphorylated NF-κB p65 (p-p65, Ser536) (Cat: 3033) and NF-κB p65 (Cat: 8242) were purchased from Cell Signaling Technology (Beverly, MA, USA). The rabbit antibodies against p53 (Cat: 345567), phosphorylated p38 MAPK (p-p38, Thr180/Tyr182) (Cat: 310091), and phosphorylated JNK (p-JNK Thr183/Tyr185) (Cat: R26311) and the mouse antibodies against p38 MAPK (Cat: 200782) and JNK (Cat: 201001) were purchased from ZENBIO Inc. (Chengdu, China). The rabbit antibodies against p21 (Cat: 10355-1-AP) and the lamin B1 (Cat: 12987-1-AP) polyclonal antibodies were purchased from Proteintech Group (Chicago, IL, USA). The polyclonal rabbit antibody against EV71 VP1 (Cat: GTX132339) was purchased from GeneTex, Inc. (Alton Pkwy Irvine, CA, USA). The rabbit antibody against EV71 3D (Cat: A8608) was purchased from Abclonal (Wuhan, China). The hydrogen peroxide (H_2_O_2_) was purchased from Guangzhou Chemical Reagent Factory (Guangzhou, China). 

### 2.3. Exosome Isolation and Purification

The cells were cultured in an exosome-depleted medium for 48 h. The cell supernatant was collected to isolate the exosomes using ultracentrifugation. Briefly, the harvested supernatant was subjected to differential centrifugation at 4 °C, starting with centrifugation at 300× *g* for 10 min, followed by centrifugation at 2000× *g* for 10 min. The pellet was discarded and the supernatant was filtered with a 0.22 μm filter (Millex-GP; Millipore, Bedford, MA, USA) to remove the remaining cells and cell debris. The supernatant was further ultracentrifuged at 120,000× *g* for 70 min using a Beckman SW41 Ti rotor (Beckman Coulter; Brea, CA, USA). The resulting pellet was washed with PBS and ultracentrifuged at 120,000× *g* for 70 min again. After discarding the supernatant, the exosome pellet was immediately resuspended in 100 μL PBS and stored at −80 °C until use. The number of exosomes was identified as previously described [16]. Briefly, the exosomes with a total protein amount ranging from 0.5 to 2.0 μg were incubated in 1 mL of medium for the treatment of the cultured cells.

### 2.4. NanoSight Tracking Analysis

The isolated exosomes were subjected to NanoSight tracking analysis (NTA) using a NanoSight NS300 instrument (NanoSight Ltd., Amesbury, UK). The solution containing the ucMSC-EXO was injected into the laser chamber using a 1 mL syringe and 60 s recordings were performed. The mean, mode, median, and estimated concentration for each particle size were analyzed. Data were processed using NTA 2.3 analytical software.

### 2.5. Transmission Electron Microscopy (TEM)

The exosome samples were dropped on a para-membrane, and a carbon-coated nickel grid was placed on the drop for 30 to 60 min. The grids were washed with 1 PBS three times and fixed in 2.5% glutaraldehyde for 30 min. The samples were then treated with 1% uranyl acetate for 30 s, rinsed 3 times with ddH_2_O, and detected using a JEM1400 120 kV TEM (JEOL Ltd.; Peabody, MA, USA).

### 2.6. Oxidative Damage and Exosome Treatment

For the establishment of the premature senescence model, the HSFs were treated with 0, 10, 50, 100, 200, and 400 μM of H_2_O_2_ in serum-free high glucose DMEM at 37 °C with 5% CO_2_ for 1 or 2 h. The cells were then washed with the serum-free medium twice to remove the residual H_2_O_2_. In the exosome treatment group, the H_2_O_2_-treated cells were washed with the serum-free medium and incubated with the exosomes for 48 h for further assays.

### 2.7. Western Blotting

The cells were harvested and lysed using an RIPA buffer (Beyotime, Shanghai, China) containing 1 mM of protease inhibitor (Roche Diagnostics; Penzberg, Germany). The total proteins were extracted and the protein concentrations were determined by using a bicinchoninic acid (BCA) Protein Assay Kit (Beyotime). For each sample, 20 μg of the total protein was loaded onto a 10–15% SDS-PAGE gel. The proteins were transferred to a polyvinylidene fluoride (PVDF) membrane (Millipore), followed by being blocked with 5% skim milk at room temperature for 1 h and then incubated with primary antibodies at 4 °C overnight. The membranes were washed with PBST and incubated with HRP-conjugated secondary antibodies at a dilution of 1:5000 (Proteintech Group) for 1 h at room temperature. The blots were analyzed using the ChemiDoc Touch Imaging System (Bio-Rad; Hercules, CA, USA).

### 2.8. Cell Proliferation Assay

For the Cell Counting Kit-8 (CCK8) assay, the HSF cells were plated at 1 × 10^5^ cells per well in 24-well plates and incubated overnight in DMEM supplemented with 10% FBS. The cell proliferation index was measured using a Cell Counting Kit-8 (Cat: CK04-500) (Dojindo Laboratories; Kumamoto, Japan) according to the manufacturer’s instructions. The absorbance was measured at a wavelength of 450 nm. 

### 2.9. Lactate Dehydrogenase (LDH) Assay

The HSF cells (1 × 10^5^) were seeded in a 24-well plate and subsequently treated with H_2_O_2_ in different concentrations. The supernatants of the HSF cells were collected and the levels of LDH were assessed using the LDH cytotoxicity detection kit (Dojindo Laboratories) according to the manufacturer’s instructions. Briefly, 200 μL of supernatant was added to each 96-well plate in triplicates. Then, 50 μL of the working solution was added and wrapped in tin foil to avoid light. After a 10 to 30 min reaction at room temperature, 25 μL of the stop solution was added to each well and mixed. The absorbance of each sample was read at 490 nm using a Varioskan LUX microplate reader (Thermo Fisher Scientific, Waltham, MA, USA).

### 2.10. Senescence-β-Galactosidase (SA-β-Gal) Staining

The HSF cells (1 × 10^5^) were grown in a 24-well plate and treated with different concentrations of H_2_O_2_ for 2 h, and subsequently treated with exosomes at different amounts for 48 h. The cells were washed with PBS and stained with an SA-β-Gal kit (Cat: C0602) (Beyotime; Shanghai, China) according to the manufacturer’s instructions. Firstly, the cells were fixed using a β-Gal fixator at room temperature for 15 min. After washing them with PBS three times, the cells were added to 500 μL of staining solution, and incubated overnight in a CO_2_-free incubator at 37 °C. An inverted microscope was used to photograph randomly selected fields to determine the degree of cell senescence.

### 2.11. qPCR Assay

The total RNA from collected cells was subjected to RNA extraction using a TRIzol reagent (Invitrogen, Carlsbad, CA, USA) and reverse-transcribed into cDNA. A quantitative real-time PCR (qPCR) test was completed using a Lightcycler 480 RT-PCR system (Roche Diagnostics; Mannheim, Germany) with a SYBR Green I Master RT-PCR kit (Roche Diagnostics). Relative quantitation was performed based on the ^ΔΔ^CT method. The primers used for the qPCR are shown in Table 1.

### 2.12. Plaque Assays

To test the antiviral ability of the ucMSC-EXO, the progeny viruses from the cell culture supernatants were harvested in preparation, and the titers were determined using plaque assay on the RD cells. The RD cells were seeded and it was ensured that the monolayers of the cells reached 90% confluence at the bottom of the wells. A series of 10-fold dilutions of the viral supernatants was infected for 2 h. 1%-agarose-containing DMEM was layered onto the infected cell monolayer at 37 °C for 2 to 3 days. The plaques of the EV71 viruses were observed by fixing them with 4% paraformaldehyde and stained for 30 min with crystal violet dye (0.5%) for 30 min at room temperature.

### 2.13. Statistical Analysis

All data are presented as the mean ± standard deviation (SD) from at least three repeated assays. A comparison between the two groups was performed using Student’s *t*-test. All statistical analyses were performed using Graph Pad Prism 7.0 (GraphPad Software, Inc.; San Diego, CA, USA). A *p* < 0.05 was considered to indicate a statistically significant difference.

## 3. Results

### 3.1. Characterization of ucMSCs and Identification of ucMSC-EXO

**Figure 1:**
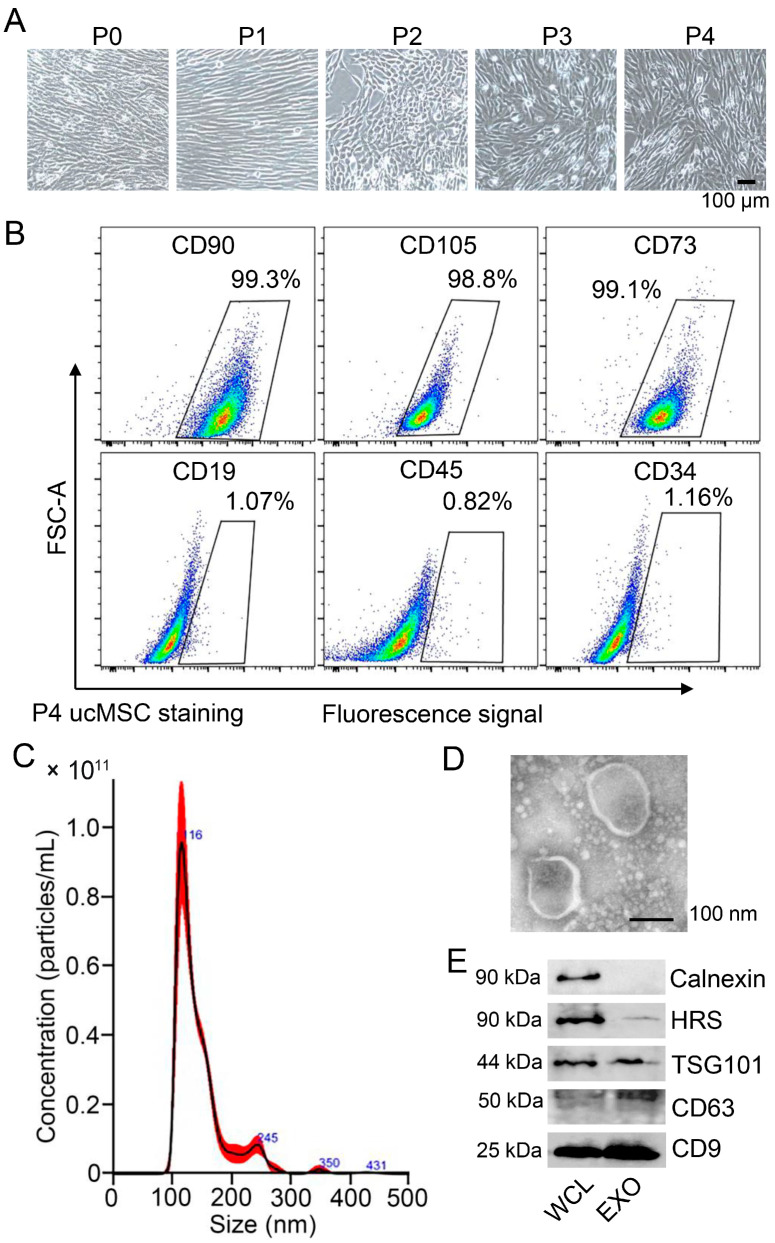
**Identification and analysis of ucMSCs and ucMSC-derived exosomes.** (**A**) Images of cultured ucMSCs from passage 0 (P0) to 4 (P4) under a light microscopy observation. Scale bar = 100 μm. (**B**) Flow cytometry analysis of surface antigens on cultured P4 ucMSCs. (**C**) NTA analysis of the isolated fractions from P4 ucMSCs supernatant. (**D**) Morphology of ucMSC-EXO under TEM observation. Scale bars = 100 nm. (**E**) Western blot analysis of positive markers (CD9, CD63, TSG101, and HRS) in exosomes. Calnexin is used as a cytosol marker. WCL, whole-cell lysis. EXO, exosomes.

The umbilical cord mesenchymal stem cells (ucMSCs) were prepared from a human umbilical cord and cultured in a serum-free MSC medium. At passage 0 of the ucMSCs, the morphological characterization of the cells was spindle-shaped with an oval nucleus in the center, and the cytoplasm protruded outward with different lengths of protrusions. After passages 1–4, the cells strongly adhered to the wall and were arranged in a homogeneous and swirled pattern (Figure 1A). The surface markers at passage 4 of the ucMSCs using flow cytometry for CD105, CD73, and CD90 were 99.3%, 98.8%, and 99.1%, respectively, whereas for CD19, CD45, and CD34, they were 1.7%, 0.82%, and 1.16%, respectively (Figure 1B), of which the positive rate was ≤5%. The phenotypic markers of the isolated cells were consistent with those previously reported for ucMSCs [17].

Next, the exosomes from the supernatants of the ucMSCs were isolated and purified. The average size and concentration of the ucMSC-derived exosomes were analyzed using NTA (Figure 1C). The ucMSC-derived exosomes were further observed as a single membrane and spheroid shape using TEM (Figure 1D). In addition, the internal content and surface markers of the exosomes, including HRS (hepatocyte growth factor receptor tyrosine kinase substrate), TSG101 (tumor susceptibility gene 101), and tetraspanins (such as CD63 and CD9), were detected in the purified exosomes (Figure 1E). Altogether, the ucMSC-derived exosomes were identified and verified.

### 3.2. ucMSC-EXO Attenuate the H_2_O_2_-Induced Oxidative Damage of HSFs

**Figure 2:**
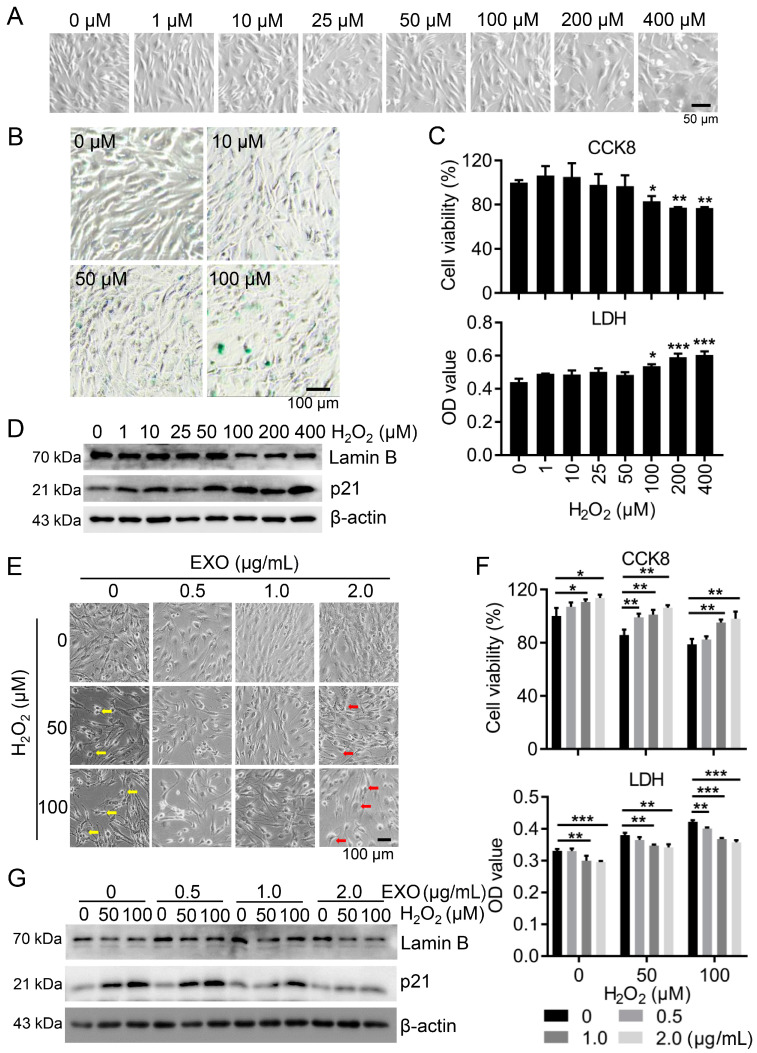
**ucMSC-EXO treatment alleviates H_2_O_2_-stimulated oxidative injury in HSFs.** (**A**) HSFs were exposed to different concentrations of H_2_O_2_ (0, 1, 10, 25, 50, 100, 200, and 400 μM) for 2 h. Morphology of cells was observed under light microscopy. Scale bar = 50 μm. (**B**) HSFs were exposed to different concentrations of H_2_O_2_ (0, 10, 50, and 100 μM) for 2 h. Cell senescence is indicated by dark SA-β-Gal intracellular staining. Scale bar = 100 μm. (**C**) Cell viability and LDH release of H_2_O_2_-treated HSFs were examined using CCK8 and LDH assay, respectively. (**D**) In H_2_O_2_-treated HSFs, the expression of lamin B1 and p21 proteins was determined using Western blotting. (**E**–**G**) HSFs were incubated with different concentrations of H_2_O_2_ (0, 50, and 100 μM) for 2 h and then treated with different amounts of ucMSC-EXO for 48 h. Morphology of cells was observed under light microscopy. The damaged cells and repaired cells are indicated by the arrows in yellow and red, respectively (**E**). Scale bar = 100 μm. Cell viability and LDH release of H_2_O_2_-treated HSFs at different concentrations (0, 50, and 100 μm) were examined using CCK8 and LDH assay, respectively (**F**). The expression of lamin B1 and p21 proteins was determined using Western blotting (**G**). Graphs are expressed as mean ± SD. *, *p* < 0.05; **, *p* < 0.01; ***, *p* < 0.001.

To establish an oxidative damage model in the HSFs, the cells were incubated with increasing concentrations of H_2_O_2_ for 2 h. Compared with a control group, the cell morphology was remarkably changed into a round shape and the number of dead cells increased with an increase in the H_2_O_2_ concentration (Figure 2A). SA-β-gal is a lysosomal enzyme that has been commonly used as the biomarker of cell senescence [18]. In parallel, we observed an increasing number of SA-β-gal-positive senescent cells after H_2_O_2_ treatment (Figure 2B), suggesting an enhanced degree of HSF aging.

Next, CCK8 assay revealed that the cell viability was significantly reduced by H_2_O_2_-induced injury, while LDH assay indicated that the degree of cell damage increased in response to H_2_O_2_ treatment (Figure 2C). To further assess the HSFs’ senescence resulting from H_2_O_2_-induced damage, both p21 and lamin B1 proteins were examined as important molecular markers for cell aging [19]. After H_2_O_2_ treatment at varying concentrations, the lamin B1 protein level was strikingly downregulated, while the p21 protein level was significantly raised after 100 μM or more (Figure 2D and Supplementary Figure S1A). These data suggested that oxidative damage enhanced cell senescence and decreased the cell survival of HSFs in exposure to H_2_O_2_ in high concentration.

To study the effect of the ucMSC-EXO on HSFs after oxidative injury, cells were treated with H_2_O_2_ and followed by treatment with the ucMSC-EXO. After treatment with H_2_O_2_, the number of senescent HSFs increased significantly, and their morphology changed significantly, including an increase in dead cells, a decrease in the overall number of cells, and rounded cell morphology, losing their original normal shape (Figure 2E), whereas these changes were lessened in cells co-treated with H_2_O_2_ and ucMSC-EXO compared to cells treated with H_2_O_2_ alone, especially at 100 μM (Figure 2E). Then, the viability and extent of the cell damage were measured using CCK8 and LDH release assay, respectively. The CCK8 assay indicated that the cell viability was decreased by H_2_O_2_ in the HSFs whereas such change was reversed by treatment with the ucMSC-EXO (Figure 2F, up). In a parallel experiment, the level of LDH in HSFs supernatants in the ucMSC-EXO groups decreased compared with control groups (Figure 2F, down). Consistently, in the control group with H_2_O_2_ alone, the lamin B1 protein level decreased while the p21 protein level increased (Figure 2G). However, the changes in the above two protein levels induced by H_2_O_2_ were reversed after ucMSC-EXO treatment (Figure 2G and Supplementary Figure S1B). Therefore, the results demonstrated that ucMSC-EXO attenuated H_2_O_2_-induced oxidative injury in HSFs.

### 3.3. ucMSC-EXO Specific Therapeutic Effects on Oxidative Damage as Indicated by Multiple Signaling Pathways

**Figure 3:**
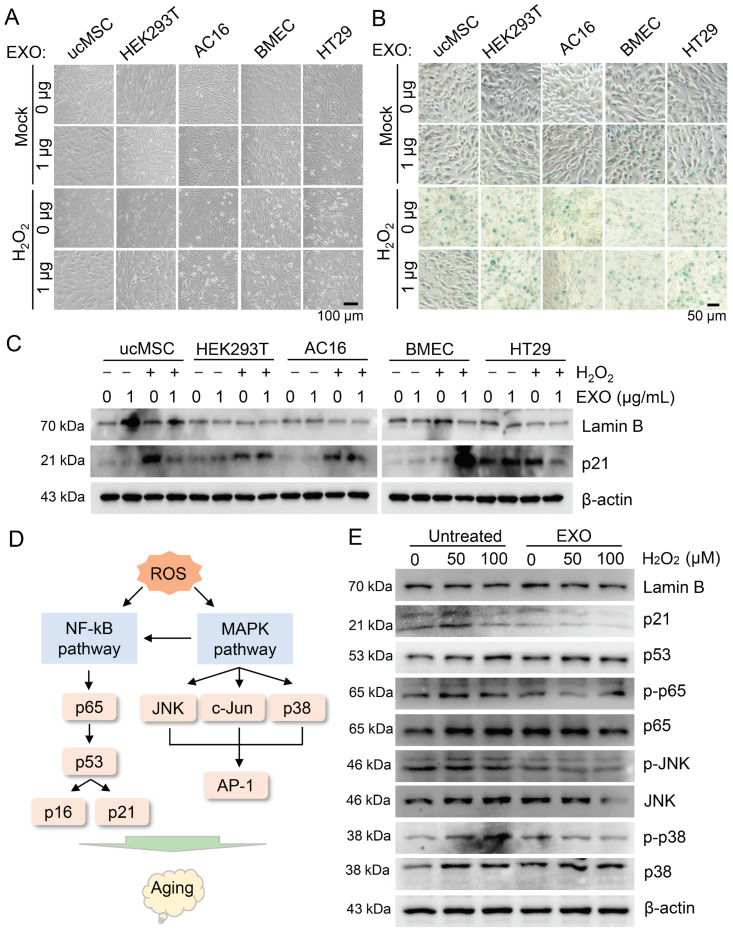
**The cell-specific ucMSC-EXO inhibit the H_2_O_2_-induced NF-κB, JNK, and MAPK signaling pathways.** (**A**–**C**) HSFs were pre-incubated in H_2_O_2_ (100 mM) for 2 h and then co-cultured with 1 μg/mL of exosomes derived from different kinds of cells for 48 h. Morphology of cells was observed under light microscopy. Scale bar = 100 μm (**A**). Cell senescence was evaluated using SA-β-Gal staining. Scale bar = 100 μm (**B**). The expression of lamin B1 and p21 proteins was determined using Western blotting (**C**). (**D**) The schematic diagram of ROS-mediated skin aging involved signaling pathways and downstream effectors. (**E**) HSFs were incubated with different concentrations of H_2_O_2_ (0, 50, and 100 mM) for 2 h and then treated with 1 μg/mL of ucMSC-EXO for 48 h. The indicated protein levels were determined using Western blotting.

To better elucidate the unique role of ucMSC-EXO in oxidative damage repair, we investigated the effects of exosomes from different types of cells on the recovery of HSFs. HSFs were treated with exosomes from ucMSCs and four other kinds of cells (AC16, HT29, HEK293T, and BMEC). In the groups with H_2_O_2_ treatment, the number of senescent HSFs significantly increased and a remarkably changed morphology was observed; however, these changes could be reduced in the presence of the exosomes derived from ucMSCs but not the AC16, HT29, HEK293T, and BMEC cells (Figure 3A). Similarly, β-galactosidase activity assays revealed that the exosomes derived from ucMSCs rather than other cells reduced H_2_O_2_-induced cell senescence in the HSFs (Figure 3B), suggesting that the therapeutic effects of ucMSC-EXO on oxidative damage of HSFs are cell-specific.

Since lamin B1 and p21 proteins are the molecular hallmarks of aging cells, the changes in the lamin B1 and p21 proteins were also seen in the HSFs in their treatment with H_2_O_2_ and cell-specific derived exosomes. In the groups with H_2_O_2_ treatment, the level of lamin B1 protein decreased, and the level of p21 protein increased, whereas these changes could be recovered in the presence of the exosomes derived from ucMSCs but not from the AC16, HT29, HEK293T, or BMEC cells (Figure 3C and Supplementary Figure S2A). These molecular and morphological observation results show that the ucMSC-EXO exerted unique oxidative damage-blocking effects in the HSFs. Thus, we identified that ucMSC-EXO specifically attenuate the H_2_O_2_-induced oxidative damage of HSFs, by downregulating the NF-κB, JNK, and MAPK signals to increase the p21 and p53 protein levels and decrease the lamin B1 protein level.

ROS accumulation impairs intracellular redox homeostasis to trigger multiple signals and downstream effectors [20]. Namely, ROS could activate MAPK and key transcription factors such as NF-κB, nuclear factor erythroid 2-like (Nrf2), JNK, and activating protein 1 (AP-1), which are also associated with a p53-mediated pathway, activating p16 and p21 and finally resulting in cellular damage and senescence (Figure 3D). To verify whether ucMSC-EXO inhibited the signaling pathways that were induced by oxidative stress, we examined the signals and target factors in the H_2_O_2_-treated HSFs. In the groups exposed to H_2_O_2_, the phosphorylation levels of p65, JNK, and p38 increased, while the level of lamin B1 protein decreased, and the levels of p21 and p53 proteins increased (Figure 3E and Supplementary Figure S2B). However, treatment with the ucMSC-EXO typically reversed these changes induced by H_2_O_2_ in the HSFs (Figure 3E and Supplementary Figure S2B), suggesting an attenuation of the NF-κB, JNK, and MAPK signaling to inhibit oxidative damage.

### 3.4. The Cell-Specific ucMSC-EXO Exert Antiviral Activity upon Enteroviral Infection

**Figure 4:**
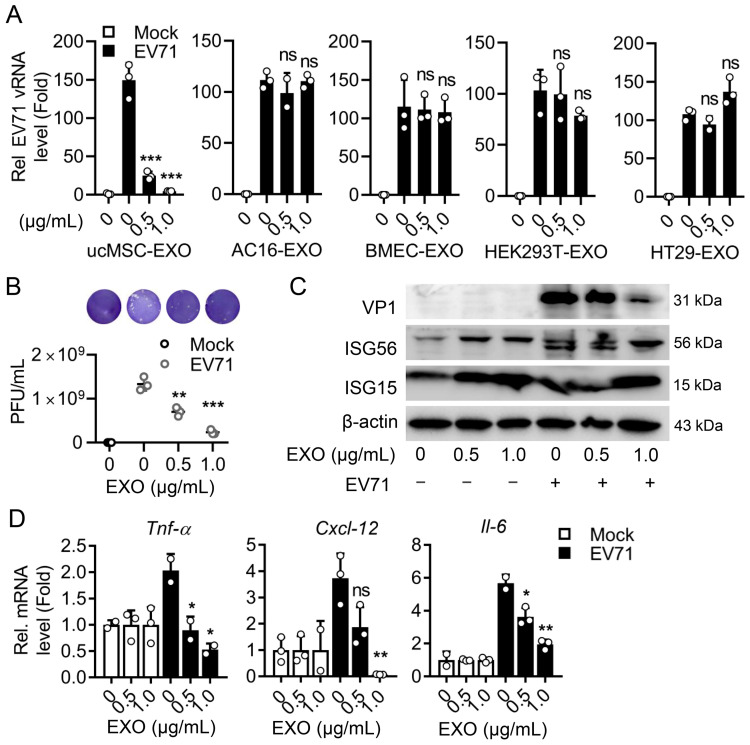
**The antiviral activity of ucMSC-EXO upon EV71 infection.** At 2 h p.i. with EV71 at an MOI of 0.5, RD cells were incubated with ucMSC-EXO at different doses (0, 0.5, 1.0 μg/mL) or the EXO from AC16, BMEC, 293T, and HT29 cells for another 12 h. (**A**) Relative expression of intracellular viral RNA levels was measured using qPCR assay. (**B**) The progeny virus titers in cell supernatants were determined using plaque assay in RD cells shown as PFU/mL. The representative images of the viral plaque assay were displayed. (**C**) Cell lysates were prepared and EV71 VP1, ISG15, ISG56, and β-actin in the cell lysates were detected using Western blot analyses. (**D**) Total RNA was extracted from the cells, and the RNA levels of *Tnf-α*, *Cxcl-12*, and *Il-6* were determined using qPCR assay. The GAPDH mRNA was used as an internal control. Data are expressed as fold change relative to control. All data are shown as mean ± SD. ns, nonsignificant; *, *p* < 0.05; **, *p* < 0.01; ***, *p* < 0.001.

To explore the antiviral activity of ucMSC-EXO, we first conducted the treatment of ucMSC-EXO in EV71-infected RD cells, an infectious model in skeletal muscle. The ucMSC-EXO and the exosomes from different cell sources were incubated at different concentrations to validate the antiviral effect. In the RD cells treated with ucMSC-EXO, the level of EV71 replication significantly decreased, while the AC16, HT29, HEK293T, and BMEC-derived exosomes did not elicit antiviral effects (Figure 4A), indicating the cell-specific nature of the ucMSC-EXO’s anti-EV71 replication effect. The culture supernatant collected after ucMSC-EXO treatment was tested using a progeny virus titer, suggesting that the production of progeny viruses was significantly reduced (Figure 4B). It is worth mentioning that the treatment of ucMSC-EXO enhanced the levels of antiviral protein ISG15 and ISG56 expression with a decrease in viral protein expression (Figure 4C and Supplementary Figure S3A). Noticeably, ucMSC-EXO treatment solidly repressed the EV71-induced expression of proinflammatory factors, including *Tnf-α*, *Cxcl-12*, and *Il-6*, (Figure 4D), suggesting an alleviation of the EV71-induced inflammatory response in the RD cells.

**Figure 5:**
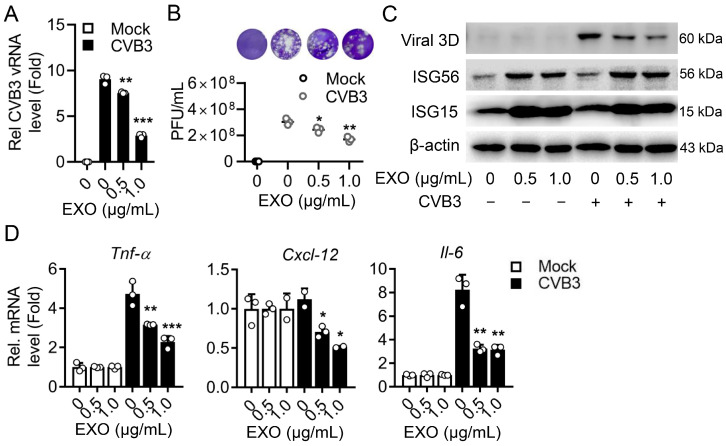
**The antiviral activity of ucMSC-EXO upon CVB3 infection.** At 2 h p.i. with CVB3 at an MOI of 0.5, RD cells were incubated with ucMSC-EXO at different doses (0, 0.5, 1.0 μg/mL) for another 12 h. (**A**) Relative expression of intracellular viral RNA level was measured using qPCR assay. (**B**) The progeny virus titers in cell supernatants were determined using plaque assay in RD cells shown as PFU/mL. The representative images of the viral plaque assay were displayed. (**C**) Cell lysates were prepared, and the viral proteins (3D), ISG15, ISG56, and β-actin in the cell lysates were detected using Western blot analyses. (**D**) Total RNA was extracted from the cells, and the RNA levels of *Tnf-α*, *Cxcl-12*, and *Il-6* were determined using qPCR assay. The GAPDH mRNA was used as an internal control. Data are expressed as fold change relative to control. All data are shown as mean ± SD. ns, nonsignificant; *, *p* < 0.05; **, *p* < 0.01; ***, *p* < 0.001.

Consistently, in another enterovirus infectious model, CVB3-infected RD cells, ucMSC-EXO significantly inhibited CVB3 replication (Figure 5A) and progeny production (Figure 5B) by means of enhancing the expression of ISG15 and ISG56 (Figure 5C and Supplementary Figure S3B), and downregulated the levels of CVB3-induced proinflammatory factors (Figure 5D). Altogether, ucMSC-EXO exhibit considerable antiviral activity upon enterovirus in muscle cells. In sum, we explore a promising insight into ucMSC-EXO as cell-free therapy both in the alleviation of oxidative damage in human skin fibroblasts and a new approach to antiviral therapy in human skeletal muscle.

## 4. Discussion

At low levels, ROS are the first line of defense and are involved in various physiological functions. However, excess ROS or insufficient endogenous defense systems impair intracellular redox homeostasis. Oxidative damage to skin fibroblasts being caused by UV irradiation and oxidant exposure, leading to skin aging, has been confirmed by a large number of studies [21,22,23]. Among them, the main cause of oxidative damage is the massive production of ROS, which breaks its balance with peroxidase in vivo. In this study, we treated fibroblasts with hydrogen peroxide to mimic the effect of amplifying ROS on skin fibroblasts. Senescent cells are usually enlarged and have a flattened shape. The nuclear envelope integrity is disrupted due to the decreased expression of lamin B1, while the DNA damage response (DDR) leads to the activation of the tumor suppressor p53, which in turn activates p21 to initiate cell cycle arrest [19]. Through treatment with different concentrations of hydrogen peroxide, we observed the morphology changes in senescent cells at 100 μM and the expressions of lamin B1 decreased and p21 increased, thus establishing a suitable model of oxidative damage in HSFs.

As one of the critical stem cell derivatives, ucMSC-EXO contain a variety of growth factors, cytokines, and RNA that improve cell function and could be used as an effective cell-free therapy in many diseases [24,25,26,27]. Here, we further investigated the therapeutic effect of ucMSC-EXO on the H_2_O_2_-induced oxidative damage of HSFs. Although previous studies demonstrate that ucMSC-EXO protect against the UV-induced senescence of HSFs [28,29], little is known about the role of ucMSC-EXO and their mechanisms of protection against the oxidative damage of HSFs. In this study, it was found that ucMSC-EXO blocked cellular oxidative damage by inhibiting multiple H_2_O_2_-activated pathways. This includes the NF-κB, JNK, and p38 MAPK signaling pathways, which increase the p21 and p53 protein levels and decrease the lamin B1 protein level. Ultimately, the ucMSC-EXO protect skin fibroblasts from being damaged by ROS.

It is acknowledged that ROS trigger the secretion of pro-inflammatory factors, leading to inflammatory damage as the concept of “inflammaging”. As such, oxidative stress and DNA damage commonly contribute to skin aging, both of which are related to inflammation [30]. Actually, the activation of JNK and p38 MAPK leads to premature senescence [30,31], implying that these pathways may be a potential link between skin aging and oxidative stress. At the same time, the oxidative stress state triggers the activation of the NF-κB pathway to regulate the expression of inflammation-related genes, resulting in the appearance of photoaging such as wrinkles and the increased thickness of the skin [32,33]. We found ucMSC-EXO inhibited these inflammation-related pathways, including NF-κB, JNK, and p38 MAPK signaling, from being activated by oxidative stress from H_2_O_2_ to protect skin fibroblasts from oxidative damage. It could be explained that ucMSC-EXO attenuate inflammation in oxidative damage repair. The potent anti-inflammatory properties of hucMSCs-EVs have been investigated in inflammatory bowel disease (IBD) [34,35], which is consistent with our findings in this study.

Since ucMSC-EXO slow down the senescence of skin fibroblasts due to oxidative damage, we also assessed the antiviral activity of ucMSC-EXO in enterovirus-infected muscle cells, and found that ucMSC-EXO can reduce EV71 and CVB3 replication and lessen the virus-induced inflammatory response. Considering the potential antiviral activity of ucMSC-EXO, the extension of the application of this nanotherapeutic strategy could be hopeful in the treatment of the concerned acute virus-associated diseases. Since the outbreak of severe acute respiratory syndrome coronavirus 2 (SARS-CoV-2), coronavirus disease 2019 (COVID-19) and the pneumonia caused by the viral infection has infected millions of individuals and caused a large number of deaths worldwide [36]. Stem-cell-derived exosomes are expected to achieve a therapeutic purpose by inhibiting viral replication and weakening the virus-induced inflammatory response in COVID-19 [37]. It is promising that ucMSC-EXO treatment is profitable for the surveillance of COVID-19. 

Innate cellular immunity constitutes the first barrier against pathogen invasion and the antiviral effect being mediated by interferons is an extremely important part of innate immunity. An interferon is a secreted protein induced by cells recognizing pathogen-associated molecular patterns (PAMPs) [38]. After IFNs are produced, they bind to the IFN receptors on the cell surface, thereby initiating the downstream signaling pathway and inducing the transcription of hundreds of ISGs. The expression of ISGs can not only directly inhibit the replication of the virus but also indirectly regulate the expression of IFNs to play an antiviral role. In our study, ucMSC-EXO treatment enhances ISG15 and ISG56 expression, leading to the inhibition of enteroviral replication and virus-induced inflammatory factor production.

ISG15 is a ubiquitin-like 17-kDa protein that is covalently conjugated to target proteins via a process called ISGylation. ISGylated 4EHP may act as a viral mRNA-specific translation inhibitor in a cap-dependent manner [39]. The IFN-induced protein with tetratricopeptide repeats (IFIT) family member ISG56 acts as a sensor to bind viral single-stranded RNA bearing a 5′-triphosphate group and plays an inhibitory role in the replication phase of the virus [40]. The antiviral effects of ucMSC-EXO could be highly related to its upregulation of ISG15 and ISG56; however, the specific mechanism by which ucMSC-EXO regulate the activation of interferon-stimulated genes is still unclear, and further verification is needed.

Future work can concern proteomics and RNA transcriptomics to verify the specific modulators in ucMSC-EXO in the performance of anti-oxidant and antiviral effects. In addition, relevant animal models or skin organoids need to be established for further verification. For example, HSFs can be cultured in an optimized three-dimensional medium for organoids [41], in which ucMSCs-EXO repair skin fibroblasts or muscle suffering from oxidative injury or viral pathogenesis as a reliable model. 

## 5. Conclusions

In summary, this study indicated that ucMSC-EXO have potential therapeutic and antiviral activities and suggested some mechanisms for these actions. These findings extend the functional exploration of ucMSC-EXO and provide potential cell-free therapeutics for anti-oxidative stress and antiviral effects on skin and muscle.

## Figures and Tables

**Table 1:** List of primers used for the qPCR in this study.

Name	Primers
*Tnf-α*	F: 5′-CTGCACTTTGGAGTGATCGG
	R: 5′-AGGGTTTGCTACAACATGGG
*Cxcl-12*	F: 5′-TCTTCGAAAGCCATGTTGCC
	R: 5′-CTTCGGGTCAATGCACACTT
*Il-6*	F: 5′-AATGAGGAGACTTGCCTGGT
	R: 5′-GCAGGAACTGGATCAGGACT
EV71	F: 5′-CTGTGCGAATTAAGGACAG
	R: 5′-GAGTTCCATAGGTGACAGC
CVB3	F: 5′-CGGTACCTTTGTGCGCCTGTT
	R: 5′-GCGGTGCTCATCGACCTGA
*Gapdh*	F: 5′-ATGTTTGTGATGGGTGTGAA
	R: 5′-ATGCCAAAGTTGTCATGGAT

F: Forward; R: Reverse.

## Data Availability

The data that support the findings of this study are available from the corresponding author upon reasonable request.

## Supplementary Materials

The following supporting information can be downloaded at https://www.mdpi.com/article/10.3390/v15102094/s1, Figure S1: Relative expression of indicated protein by Western blot analyses in Figure 2; Figure S2: Relative expression of indicated protein by Western blot analyses in Figure 3; Figure S3: Relative expression of indicated protein by Western blot analyses in Figure 4.

## Abbreviations

ROS	Reactive oxygen species
ucMSC	Umbilical cord mesenchymal stem cells
ucMSC-EXO	Exosomes derived from ucMSCs
HSFs	Skin fibroblasts
MAPK	Mitogen-activated protein kinases
JNK	c-Jun N-terminal kinase
NF-κB	Nuclear factor kappa-B
EV71	Enterovirus 71
CVB3	Coxsackievirus B3
ISG15	Interferon-stimulated gene 15
DDR	DNA damage response
SASP	Senescence-associated secretory phenotype
p38 MAPK	P38 mitogen-activated protein kinases
HFMD	Hand-foot-and-mouth disease
EVs	Extracellular vesicles
HRS	Hepatocyte growth factor receptor tyrosine kinase substrate
TSG101	Tumor susceptibility gene 101
Nrf2	Nuclear factor erythroid 2-like
AP-1	Activating protein 1
IBD	Inflammatory bowel disease
SARS-CoV-2	Severe acute respiratory syndrome coronavirus 2
COVID-19	Coronarius disease 2019
PAMPs	Pathogen-associated molecular patterns
IFIT	IFN-induced protein with tetratricopeptide repeats
hBMEC	Human brain microvascular endothelial cell
TEM	Transmission electron microscopy
PVDF	Polyvinylidene fluoride
LDH	Lactate dehydrogenase
SA-β-Gal	Senescence-β-Galactosidase
CCK8	Cell Counting Kit-8
