# Supplementary material for: Umbilical Cord Mesenchymal-Stem-Cell-Derived Exosomes Exhibit Anti-Oxidant and Antiviral Effects as Cell-Free Therapies
Source: Viruses. 2023 Oct 15;15(10):2094. doi: 10.3390/v15102094 (PMC10612094; doi:10.3390/v15102094)
Supplement: Supplementary file 1 [file viruses-15-02094-s001.zip › viruses-2596263-supplementary.pdf]

Supplementary Materials

Supplementary Figure 1

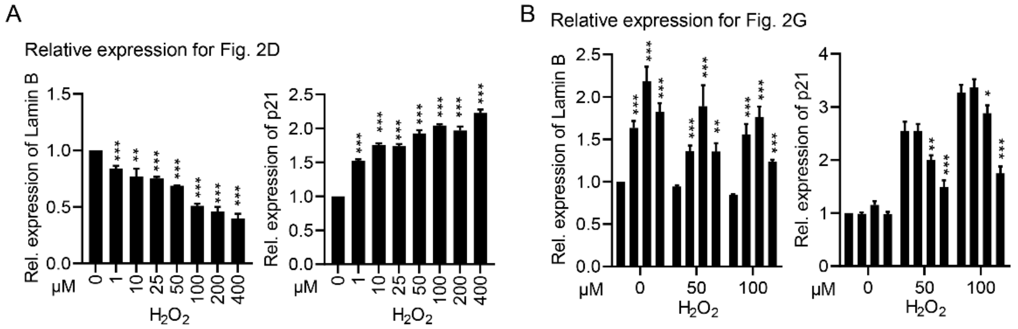

Supplementary Figure 2

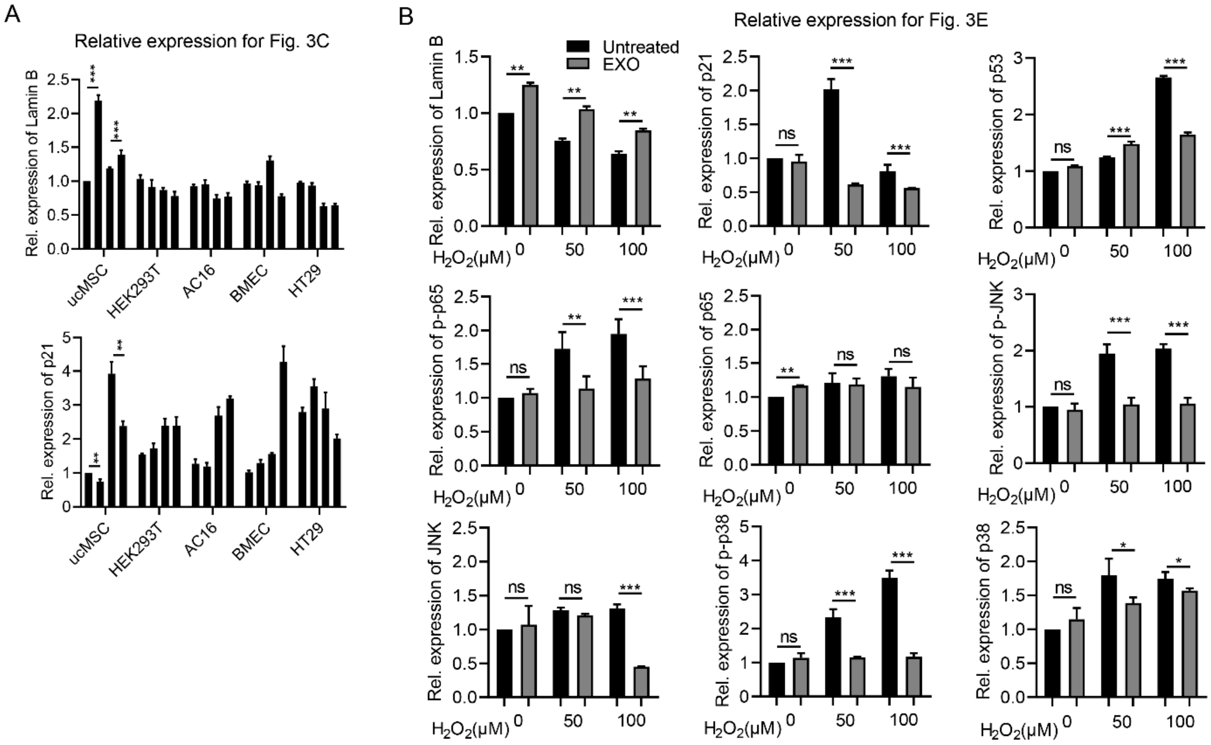

Supplementary Figure 3

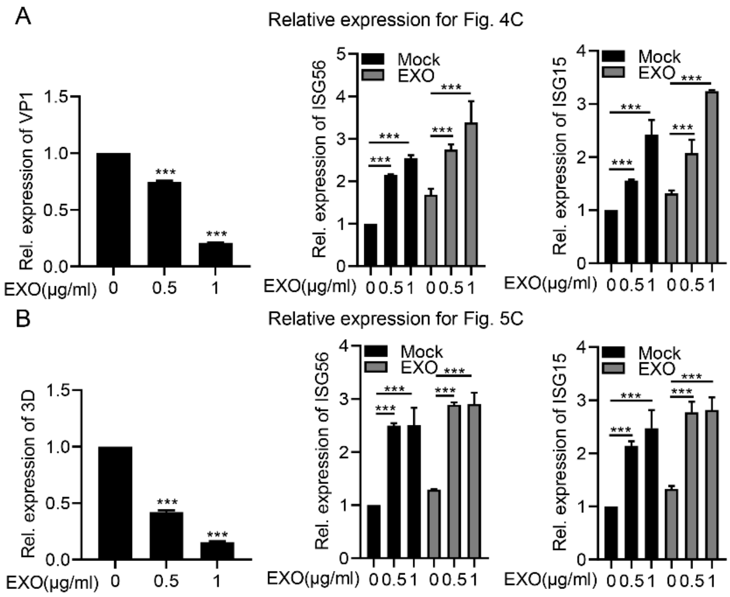

Supplementary Figure 1-3. Relative expression of indicated protein by Western blot analyses. Protein expression relative to internal control in each figure is quantified using Image J software. Data are expressed as mean ± SD. ns, nonsignificant; *, $P < 0.05$; **, $P < 0.01$; ***, $P < 0.001$. Statistical significance was determined by a Student's *t*-test.
